# Ultra-Orthodox Parents’ Perceptions of Arts Therapies for Their Children

**DOI:** 10.3390/children9101576

**Published:** 2022-10-18

**Authors:** Lali Keidar, Sharon Snir, Dafna Regev, Eliav Keidar

**Affiliations:** 1 School of Creative Arts Therapies, University of Haifa, Haifa 3498838, Israel; 2 Department of Art Therapy, Tel Hai College, Tel Hai 1220800, Israel; 3 The Interdisciplinary Research Center for Arts and Spirituality: Therapy, Education and Society, Tel Hai College, Tel Hai 1220800, Israel; 4 The Emili Sagol Creative Arts Therapies Research Center, University of Haifa, Haifa 3498838, Israel; 5 Faculty of Social Work, Ashkelon Academic College, Ashkelon 78211, Israel; 6 School of Social Work, Hadassah Academic College, Jerusalem 9101001, Israel

**Keywords:** arts therapies, ultra-Orthodox Jews, children, intercultural therapy, parents’ perceptions

## Abstract

Studies have underscored the complexity of psychotherapy for Ultra-Orthodox Jews, and cross-cultural therapy in particular, which evokes fear of disruption of basic values. Parents’ sense of responsibility for their child’s religious education exacerbates these problems in child therapy. However, there is scant research on child therapy for the Ultra-Orthodox, especially in the field of arts therapies. The present study examined the perceptions of 17 Ultra-Orthodox parents whose children were receiving arts therapies (including art therapy, dance/movement therapy, music therapy, psychodrama and bibliotherapy). Semi-structured interviews were conducted with the parents and analyzed based on the principles of Consensual Qualitative Research. The study covered five domains: (1) The parents’ experiences in therapy; (2) The parents’ perceptions of the child’s experiences in therapy; (3) Implications of environmental-social factors on the parents’ perceptions and experiences of therapy; (4) Effects of intercultural aspects on therapy; (5) Perceptions of the use of the arts in therapy. The findings show that the experiences of ultra-Orthodox parents in the arts therapies of their children is complex due to the influence of the socio-cultural context, which involves dealing with stigma and tensions in their relationship with the education system. This context also shapes their perceptions of therapy, which can be characterized as purpose-oriented. The findings also highlight the parents’ challenges in coping with the intercultural therapeutic relationship, and emphasizes the parents’ preference for a therapist from a similar religious/cultural background and for cultural supervision of therapy. However, the results also suggest that there are benefits inherent to intercultural therapy in general and arts therapies in particular, including a sense of security, openness and acceptance of the parents and children.

## 1. Introduction

Studies have underscored the effectiveness of arts therapies in treating a variety of difficulties [1,2], and suggest that arts therapies for children promote quality of life, reduce anxiety, and lead to improvement in self-perception, problem-solving skills, and emotional and behavioral problems [3]. In Israel, many arts therapists are employed by the education system [4] and most treatments provided through this system are delivered by arts therapists [5]. The primary reasons for referring children to art therapy in the educational setting are disruptive behavior, trauma, emotional difficulties and anxiety [6].

Despite the greater interest in arts therapies among the Jewish ultra-Orthodox in recent years [7], there is scant research on these therapies in general and in ultra-Orthodox children in particular. The few studies that have examined arts therapies for ultra-Orthodox children [7,8,9] have all focused on the therapists’ perspective. To the best of our knowledge, there are no studies dealing with the perspectives of ultra-Orthodox parents with respect to arts therapies for ultra-Orthodox children. Therefore, to expand and deepen current understandings of the complex phenomenon of arts therapies in ultra-Orthodox children from another point of view, and given the importance of parental involvement in child therapy [10], the present study examined the perceptions of ultra-Orthodox parents with respect to arts therapies with their children.

### 1.1. Ultra-Orthodox Jews

Ultra-Orthodox Jews constitute a distinct minority group in Jewish society in Israel and around the world, and are perceived as the most religious-conservative faction in Judaism [11]. As of 2021, the ultra-Orthodox population in Israel numbered about 1,226,000, constituting 12.9% of the total population. The ultra-Orthodox Jewish population in the world is estimated at 2,100,000, which is about 14% of the Jewish population in total. Most ultra-Orthodox Jews live in Israel. The second largest concentration is in the United States followed by the United Kingdom, Canada and other countries [12]. The ultra-Orthodox population is characterized by the fastest growth rate in developed countries, as a result of their young age at marriage and numbers of children. For these reasons, half of the Israeli ultra-Orthodox population are under 16 years old [13]. Ultra-Orthodox society is traditional, collective and patriarchal, and emphasizes faith in God and strict commitment to Jewish law, along with loyalty to the community and obedience to rabbinic authority [11,14]. It consists of three main factions: Hasidim, Lita’im (Lithuanians) and Sephardic Haredim, which differ in terms of their customs, leadership, educational institutions, appearance and other factors [15]. The Hasidim and the Lita’im are the two main factions in the ultra-Orthodox population in Israel, and most are of European descent. The Lita’im emphasize the value of learning the Torah more than the Hasidim, and consider excellence in religious studies to be a central criterion for determining social status. Lita’im are more modern in their lifestyles, clothing and occupations. In contrast, the Hasidic view emphasizes the hidden spiritual dimensions of reality. Unlike the Lita’im, many Hasidim speak Yiddish and start working for a living at an earlier age. Sephardic Haredim trace their origins to the Eastern Mediterranean [16]. Another group is composed of Baalei Teshuva; in other words, people who have shifted from a secular lifestyle to a religious/ultra-Orthodox lifestyle. Despite their opposition to a secular way of life [11], in the last two decades there has been a turning point in the integration of the ultra-Orthodox into Israeli society, which is reflected in the growing presence of ultra-Orthodox men and women in higher education and the labor market [13].

### 1.2. Ultra-Orthodox Children

Children are perceived as a tremendous blessing in the ultra-Orthodox community, and birth is the main goal of marriage [14]. Due to the rigid division of gender roles in ultra-Orthodox society, there are differences in the relationships between ultra-Orthodox fathers and mothers and their children, such that mothers tend to be responsible for the emotional aspects of raising their children while fathers focus on the intellectual and spiritual aspects [17]. Daughters become partners in child raising, in that they take care of their younger siblings from early childhood, and the entire community provides support to parents [18]. The ideal for ultra-Orthodox boys is to become “Talmidei Chakhamim” (students of sages, who are well-versed in Jewish law), while girls are expected to support and enable them to fulfill their vocation [18,19]. The self-identity of ultra-Orthodox children derives its meaning largely from belonging to the community [20]. Children and adolescents in the ultra-Orthodox community receive clear instructions as to what is allowed and what is forbidden, and they are expected to follow them [21], to constrain confusion, uncertainty, identity crises, search and self-discovery [22]. Compared to secular children, ultra-Orthodox children are required to display more maturity, independence and responsibility [23], and are expected to shape their desires according to God’s will and commandments [22]. Norms, values and customs are conveyed to children through everyday rituals, symbols and ceremonies that do not leave much space for self-expression. Cultural values and norms are woven into children’s lives through stories, songs and games, which are a means of educating and socializing ultra-Orthodox children and strengthening their sense of belonging to their community [18,20].

### 1.3. Challenges in Therapy with Ultra-Orthodox Jews

In most cases, ultra-Orthodox adults and children are treated by arts therapists who are not ultra-Orthodox (a secular therapist or a therapist in another stream of Judaism), because there are few ultra-Orthodox graduates of academic training tracks in therapy. Thus, ultra-Orthodox therapy can be defined as intercultural therapy, where the intercultural differences refer not only to differences in ethnic background, but also to differences in religious affiliation [24]. Thus therapy also involves an epistemological difference between the traditional-collectivist value system that underlies the culture of ultra-Orthodox clients, and the Western value system on which psychotherapy is grounded [25]. Intercultural therapy involves dealing with differences in culture-bound values, socio-economic status and biases related to language and communicative style. Culturally diverse clients do not share most of the values and characteristics rooted in the goals and processes of therapy, and they may differ or be limited in emotional expressiveness, self-disclosure, openness and norms of intimacy [26]. Although there is now greater acceptance of psychotherapy and outside help, especially for children at risk [27], studies show that the intercultural gap still impacts the ultra-Orthodox encounter with psychotherapy and creates significant conflicts [28]. For example, ultra-Orthodox clients may experience a conflict between dealing with difficulties privately and autonomously within the community and seeking help from external sources [11], difficulty opening up about issues that are not culturally acceptable [29], expecting practical solutions that are limited to what is allowed and forbidden according to culture and religion [30], and fear of stigma that may lead to negative consequences for future matchmaking of the individual and other family members, the ability to get a job in the community, social ties, and integration in educational institutions [31,32]. In child therapy, these difficulties can be intensified for the ultra-Orthodox client’s parents, who feel a sense of responsibility for the child’s religious and spiritual education [33].

The few studies that have examined arts therapies for the ultra-Orthodox have noted other challenges that arise in this form of therapy. For instance, in a qualitative study based on interviews with 14 ultra-Orthodox art therapists and ultra-Orthodox adult clients, the interviewees described the clients’ fear of forming a close relationship with the therapist. This was experienced as a childish need, leading to feelings of self-blame and to responses aimed at calming anxiety and blurring closeness, which prevented the development of the therapeutic relationship. The findings also indicated that clients found it difficult to share past experiences, failures, or negative feelings towards the therapist, and repressed negative emotions out of a desire to maintain confidentiality [34]. In another qualitative study which involved interviews with 17 non-Haredi arts therapists, the therapists described the difficulties experienced by ultra-Orthodox children in regulating emotional release, which was manifested in swings from restrained and held conduct to over-ramping resulting from a sense of emotional flooding. This study also highlight the predominant fear of exposure in therapy: children refrained from talking about their families and their parents avoided reporting essential content and sometimes even switched to ultra-Orthodox therapists [7]. In general, the participants emphasized that the encounter between art and religion/culture was complex and raised concerns about violating religious prohibitions. This led to careful avoidance of anything related to sexual content or playfulness [7,34]. In cases where the therapist was secular, there was fear that the treatment outcomes would be inconsistent with the ultra-Orthodox worldview. Thus, clients tended to screen the therapist’s lifestyle and perceptions [34], which at times led to hesitation in establishing a therapeutic relationship or simply to dropping out [7]. However, the cultural gap can at times increase clients’ sense of comfort and openness given the negligible likelihood of disclosure of complex issues to the ultra-Orthodox community [7,8]. The studies described above also indicate that arts therapies can provide ultra-Orthodox clients with an indirect and less threatening way to express content that cannot be talked about elsewhere and a means of self-disclosure [7,8,34]. A case study on art therapy for an ultra-Orthodox child provided by a non-Orthodox therapist noted that the potential benefits of art therapy for ultra-Orthodox children include the possibility of expressing negative emotions in an indirect way, which reduces the children’s fear of violating cultural norms, religious laws or tarnishing their good name. The author suggested that art therapy can give the ultra-Orthodox child some modicum of freedom of choice, beyond what is authorized in the community [8].

### 1.4. Parents’ Perceptions of Their Children’s Therapy

Various studies have focused on examining parents’ perceptions of therapy for their children, since child therapy depends on the parents’ consent, based on their assessment of the severity of the child’s condition and the physical, economic and attitudinal barriers that may prompt parents to reject therapy [10]. A quantitative study that examined perceptions and attitudes toward therapy of 194 African American and Caucasian parents found that parents who perceived fewer barriers and had more positive attitudes toward receiving therapy for themselves also manifested more positive attitudes toward therapy for their children [35]. A quantitative study that examined the perceptions of 122 mothers from different ethnic backgrounds (European-American, African American and Latino) found that mothers’ fears of being forced to do or say things against their will and their fear of criticism predicated their intentions to reject therapy for their children [36]. Another quantitative study, in which 405 parents filled out questionnaires as part of an outpatient treatment service for children’s aggressive and antisocial behaviors, found that parents’ expectations predicted barriers to participation in therapy and dropping out of therapy prematurely, and were influenced by socioeconomic status, belonging to a minority group, and parental stress [37]. A quantitative study on parents of 222 children with increased anxiety symptoms [38], as well as a qualitative study in which six mothers of children with behavioral disorders were interviewed [39], reported that fear of stigma and negative consequences was a significant barrier to the decision to authorize therapy for their children [38,39]. In terms of gender differences, in a quantitative study of 89 Indian parents, mothers reported greater openness to therapy for their children than fathers [40].

Few studies have examined the perceptions of ultra-Orthodox parents, all of which have implemented a qualitative approach and mainly used interviews. These works have focused on examining the perceptions of ultra-Orthodox parents towards child-raising as related to their mental well-being (including parental love and corporal punishment) [41,42], and their perceptions and reactions to situations of risk and harm to children [43], such as a study that examined drawings and the short narratives of 21 ultra-Orthodox mothers on the topic of publicly revealing their children’s sexual abuse [44]. Some of these studies suggest that the differences in perceptions between the ultra-Orthodox parents and the non-Orthodox professionals may have affected their relationship and led to parental fear of possible dangers of the intervention and a preference to get help from within the community [42,43]. The study that has come closest to examining ultra-Orthodox parents’ perceptions of therapy interviewed 21 ultra-Orthodox mothers and fathers in Antwerp, Belgium on their help-seeking behaviors. The findings showed the importance the parents attributed to the educational system, their preference for non-conventional treatment, and their fear of labeling and spiritual erosion [33]. However, no studies have been conducted on the perceptions of ultra-Orthodox parents towards arts therapies for their children. Since the few studies that have examined arts therapies in ultra-Orthodox children have dealt with the therapists’ point of view alone, and in light of the importance of parental engagement in child therapy [10,36,37], the current study explored the following research question: How do ultra-Orthodox parents perceive arts therapies for their children? 

## 2. Materials and Methods

### 2.1. Participants

Seventeen ultra-Orthodox parents whose children were receiving arts therapies participated in this study (12 mothers and five fathers). The parents ranged in age from 29 to 54; one parent did not provide information on his age (*M* = 37.5, *SD* = 7.13). Most lived in northern Israel in the Haifa region (13 parents), and the remainder lived in the center of the country (two parents), the Jerusalem area (one parent), or in the south (one parent). The sample represented all the ultra-Orthodox streams, including Hasidim (seven parents), Lita’im (seven parents), Sephardic Haredim (one parent) and Baalei Teshuva (two parents). The parents had between 1 and 8 children (*M* = 5, *SD* = 1.81). Three of the interviewees had more than one child receiving therapy (2–4 children in treatment).

The 13 boys and nine girls in therapy ranged in age from 3 to 14 at the time they started therapy (*M* = 7.3, *SD* = 2.74); the age of one child was not indicated. The duration of therapy for each child ranged from 6 to 60 months (*M* = 24.5, *SD* = 14.8). They were receiving visual art therapy (10 children), dance/movement therapy (four children), bibliotherapy (four children), music therapy (two children) and psychodrama (two children). Therapy took place in an ultra-Orthodox institution (19 children), a school (one child), a public clinic (one child) and private clinic (one child). Of the therapists who provided arts therapies for the children, 12 were secular, seven were Religious-Zionist and three were ultra-Orthodox (see Table 1 for general data, designed to preserve anonymity).

According to the parents’ reports, the children were referred for one or more of the following problems: emotional problems manifested in frustration, low self-confidence and anxiety (11 children), behavioral problems including outbursts of anger, difficulty in accepting boundaries and violence (10 children), social problems including maladaptive social behavior and loneliness (seven children), learning disabilities (four children), ADHD (three children) and communication disorders (two children). The referrals were made on the parents’ personal initiative (eight parents), the educational system (five parents), or professionals such as psychologists, family doctors or social workers (three parents).

### 2.2. Procedure and Ethics

Arugot is a therapy center for ultra-Orthodox children located in the north of Israel, run by members of the ultra-Orthodox community and rabbinic figures, although most of the arts therapists are not ultra-Orthodox. The Arugot Institute staff contacted parents whose children were receiving arts therapies at the institution. Parents who expressed interest in participating in the study contacted the first author. These parents received explanatory and consent forms via e-mail, in which it was clarified that they were not obligated to participate in the study, and that they could withdraw at any stage, without repercussions on their children’s therapy in any way. They were also guaranteed that their identity would remain confidential throughout the stages of the research and the publication of the results. Thirteen parents agreed to be interviewed. Simultaneously, an ad was posted on WhatsApp to specific groups. This led to the recruitment of four more parents who contacted the first author for further details. These parents also received explanatory and consent forms via e-mail. The interviews took place between October 2020 and December 2020. Each interview lasted about an hour. Due to the outbreak of COVID-19, all interviews were conducted over the phone. All parents received the equivalent of $30 as compensation for their time. The interviews were recorded and transcribed after securing the participants’ consent. All identifying details were deleted after the interviews were transcribed, and the recordings were destroyed after the transcription. This study was approved by the Ethics Committee of the Faculty of Social Welfare and Health Sciences at the University of Haifa (278/19). The approval was first received on 28 July 2019 and updated on 19 December 2020.

### 2.3. Data Collection

Semi-structured in-depth interviews with the mothers were conducted by the first author, who is a woman, and with the fathers by the fourth author, who is a man, to foster comfort and openness among the interviewees, and out of sensitivity for the gender division that is the norm among the ultra-Orthodox. The interviewers used an interview guide composed of open-ended questions (see Appendix A). The parents were asked about their perception of the arts therapies their children were receiving. The interviews covered three main foci: (1) The parents’ experiences of their child’s therapy: the goals of the therapy, core components, central dilemmas, implications for the decision to seek treatment; (2) perception of arts therapies: the role and qualities of the arts in therapy, the relationship between the arts and culture and religion; (3) intercultural therapy: the importance of the cultural/religious background of the therapist, the effect of this background on the therapy, advantages and disadvantages of similarities and differences in culture. The interview guide was formulated based on the literature, as well as our experience of the research context [45]. Drawing on a previous study in which arts therapists working with ultra-Orthodox children were interviewed [7], the interview guide was adapted for interviews with ultra-Orthodox parents. To ensure that the questions were formulated asked in a non-biased and non-leading way, descriptive and comprehensive questions were used. Then, to gain a more in-depth understanding, probes were used, such as requests to give an example and rephrasing of the participants’ responses for confirmation.

### 2.4. Researchers’ Lenses and Biases

All four authors are therapists: the first three authors are art therapists and the fourth author is a social worker. The second and third authors are secular. Both interviewers—the first author and the fourth author—identify as Religious-Zionists with no previous personal or professional relationships with the participants. The religious/cultural affiliation of the interviewers was not explicitly stated to the interviewees, but was indicated if asked. Nevertheless, it can be assumed that the religious affiliation of the interviewers had an impact on the exchange, due to the natural use of shared concepts and expressions. The range of observance of the authors provided a balance, where the more observant authors could provide grounding in concepts without a cultural affiliation which could have biased the interpretation, whereas the secular authors could provide a more external perspective on the findings. All the authors were thus cognizant of the possible impact of their own lived experiences, which helped ensure results that were not biased. Although the authors’ cultural/religious backgrounds were different, they approached the interviewees and the data analysis process with an awareness of intercultural differences, with the excitement that accompanies such an encounter, curiosity to hear and learn, and with the desire to understand the participants’ experiences while striving for cultural humility [46].

### 2.5. Data Analysis

The research method and data analysis adhered to the principles of Consensual Qualitative Research (CQR), which is based on phenomenological elements whose purpose is an in-depth observation of the experiences and subjective perceptions of the participants, while striving for a consensus by a team of researchers [47]. The method was developed in the field of psychotherapy research [48], and is therefore common in research on counseling and therapy processes [49,50]. This method is considered very effective in researching topics that have not yet been studied or that are not sufficiently theoretically based [36]. Therefore, this method is used in relatively new research fields such as arts therapies [51], and in particular in the study of arts therapies for the ultra-Orthodox [7].

In the first stage of data analysis, three interviews were analyzed separately by the first three authors, who are researchers and art therapists, to identify the central domains that emerged from the data in each of the interviews. Then, the three authors met to reach a consensus on the definition of the central domains of the three interviews (cross-analysis). Subsequently, the first author analyzed the rest of the interviews, by dividing them into the agreed domains and making adjustments for new material if necessary. In the next stage, the researchers went through the material associated with each domain separately, defined the core ideas in each domain, and then they met for a discussion to reach a consensus. The prevalence of the core ideas in the Results section is characterized as follows: the term “most parents” is used to describe a phenomenon that was identified in over 75% of all interviews, that is, in 13 or more parents; the term “some parents” describes a prevalence in 25–75% of all interviews, that is, between five to 12 parents; and the term “a few parents” describes a prevalence of less than 25% of the cases, that is, four parents or less [47].

### 2.6. Trustworthiness of the Study

Since transparency is a significant aspect of trustworthiness, the research process is reported in detail, so that the process can be replicated in other studies [52]. The relevant background on the authors was provided above. Because ensuring credibility is particularly important for establishing trustworthiness, the authors furthered their prior acquaintance with the culture of the participants and, as mentioned, an established research method was chosen, that is appropriate for the study of the phenomenon and has been used previously in studies of a similar nature [53]. In order to increase credibility, investigator triangulation was also carried out, in the form of data analysis in a team [52]. For an honest response from the participants, it was made clear to them that their sincere position was important and that there were no right or wrong answers in the study. As mentioned, there was a gender match between the interviewer and the interviewee to contribute to feelings of comfort and openness. The interviewees were given the right to refuse participation and withdraw at any stage, and it was made clear to them that their privacy would be fully preserved so that they should not feel afraid to express themselves, and that the information they provided would be as reliable as possible [53]. Conducting the interviews over the phone helped increase reliability, as this technique enabled the participation of individuals belonging to different factions in ultra-Orthodox society, who most likely would not have participated if it had been a face-to-face interview, which would have resulted in narrowing the population participating in the study [54]. The questions asked were open-ended while avoiding the expression of attitude, opinion or presupposition, so that the interviewees would tell their story in a way that was not biased or dictated [55]. However, we decided against member checks to avoid bothering the participants, whose access and recruitment were complex.

## 3. Results

### 3.1. The Parents’ Experiences in Therapy

#### 3.1.1. The Parents’ Relationship with the Therapist Was Seen by Them as Important

The findings indicated that most parents understood the importance of their relationship with the therapist and perceived it as a key to the success and continuation of therapy: “You cannot separate yourself from it. It will not work. Must cooperate”. As for their expectations from their relationship with the therapist, some of the parents emphasized that their main goal was to understand the therapeutic process and the child who was receiving therapy. These parents hoped to better understand how the therapist sees and understands what is happening in therapy and its continuation, as well as the reasons for the child’s challenging behaviors and inner world: “A little explanation of mental activity, I’m very interested in what causes what, and why it happens”. In addition, a few parents said they wanted practical tools from the therapist on ways to cope, especially in difficult situations: “How to deal with conflicts that arise all the time, how to manage them”.

The descriptions of some of the parents suggested that although both spouses were involved to some extent in the relationship, the mothers were more so. Some parents received parental guidance, and a few called the therapist on the phone in times of crisis, and took part in dyadic therapy. Most parents’ expectations concerning their relationship with the therapist appear to have been met. Some parents talked about the guidance they received from the therapist about their conduct at home and with the child, which included instructions and concrete tools for coping. They also described the explanations the therapist gave them about their child’s behavior and the therapy process. Some parents emphasized that rather than getting advice, the sessions with the therapist often involved providing more information about the child’s life: “She devoted a lot of time to the things I feel because I am with him most of the time, and sometimes we know what is good for the child and what is not”.

#### 3.1.2. Along with the Parents’ Positive Feelings towards the Therapist, Ruptures Sometimes Appeared in the Parents’ Relationship with Her

Some parents brought up things that impeded their ability to be involved in the relationship with the therapist, including the burden of raising children and having to work, the financial strain caused by the cost of the meetings and COVID-related social distancing measures. Despite their positive perception of the therapist, including a strong sense of trust, the therapist’s professionalism and empathy, containment, devotion and avoidance of judgment, at times there were conflicts. For example, even in cases where a good relationship had developed between the parents and the therapist, they sometimes found it difficult to follow the steps that had been suggested in parental guidance: “It was something that was between me and her all the time, she told me to loosen up and I was constantly stressed”. Some of the parents criticized the therapist. They felt they were being disrespected as parents, that they were being watched, or pushed too much: “I felt really bad there. I felt I have no connection, I don’t understand her, she asks about things that are difficult to talk about and she doesn’t understand that”. These same parents criticized the therapist’s approach and avoidance of in-depth work that could include a painful reflection for the child: “I wanted to tell her—deal with it, like, you’re the therapist. I think she gave up to him”. A few parents requested termination and referral to a different therapist, which they considered a trivial move: “I asked for a replacement. Nothing happened”.

#### 3.1.3. The Parents Expected Perceptible Results but Some Understood That This Is a Complex Process

Some parents attached considerable importance to the success of therapy, and wanted the therapy to lead to concrete results, including targeted behavioral improvement (such as better discipline and respect of boundaries, the ability to delay gratification and less anxiety), as well as more self-confidence, self-expression and flexibility. These parents wanted quick results and explained that simply having their child enjoy therapy was not enough; rather, significant work should be done and that the sessions should be well-utilized: “It’s important to me that they don’t waste their time. It’s important to me that the therapy be exhaustive”. These parents expressed frustration when they felt that the therapy had stalled and was not leading to noticeable progress, and at times terminated therapy if there were no tangible results after a plateau period: “Of course you don’t expect 100% success at first. You go to the five or six next sessions. If there is an improvement then you say—Okay, it’s worth continuing. If not, no”. At the same time, some parents stated that although they aimed for results, they came to realize that this process would not necessarily lead to “correction” of the difficulty: “It’s a process. It’s something you understand more and more over time. On the one hand, you want results in the here and now, but it doesn’t work. It doesn’t make sense either”.

To make therapy a success, some parents said that they encouraged their children to share their worlds and feelings, out of the understanding that this is the only way therapeutic work can be done: “If you don’t really come to work and share, then why are you here? It’s a waste of time. There won’t really be work on the essential needs”. Some parents explained that for therapy to be a success, they avoided interfering in the therapy process, and did not impose restrictions on content allowed in therapy, even if this was not to their liking. Only one mother described a significant intervention in therapy, including restrictions on conversation and a requirement that the therapist’s suggestions be approved by the parents: “I told her what she could talk about and what not”.

#### 3.1.4. Parents Felt That Therapy Enhanced Their Understanding of the Child and Their Parenting, and Led to an Improvement in Family Relations

Some parents described how the therapy contributed to their understanding of the child, the reasons underlying behaviors and the best ways to deal with this child. In their view, this made their dealings with the child easier and improved their relationship: “Thanks to therapy I began understand my son. I saw him differently. And then I had a much easier time with him”. A few parents stated that the change in their conduct as parents and the advice they had been given led to an improvement in their lifestyle at home and had a positive effect on the relationship between all family members: “It contributes a lot to the building of the bond between the brothers, to the bonds between the parents and the children. Our perspective as parents is different”. A few parents discussed the realization that they were contributing to their child’s challenging behavior, and the need to go through a process of change themselves: “You first need to address the root of the problem. When the tree is sick, if you treat the branches and the root is still diseased it will not help”. However, despite understanding their effect, and even in cases where the therapist recommended that the parents consider therapy themselves, a few parents admitted that they did not intend to do so: “They wanted us to go to therapy as parents. But we already have too much to do, we don’t have the energy”.

#### 3.1.5. Most Parents Perceived the Therapy as Effective, although Some Were Dissatisfied

Most parents stated that there had been improvement after therapy. Some parents only described a partial improvement—“really small, minor benefits”, whereas others reported an improvement which only emerged gradually over time: “Sometimes it doesn’t seem that impactful, but over time you realize that it works”. Certain parents described a substantial improvement: “She saved the girl. She supported her in an unusual way”. However, a few parents stated that despite the improvement, the initial reason for consulting was only partially resolved or at times remained unresolved: “Shall I say there is no problem? Has this been fixed? Is it behind us? Certainly not”. Similarly, a few parents were unsure why the improvement had taken place and did not necessarily ascribe it to therapy: “I don’t know if I can attribute it to the therapy or if he just got older”. A few parents stated that they could not point to any change that occurred in their child as a result of therapy.

Mixed perceptions also appeared in the parents’ satisfaction with arts therapies. A few parents stated that they were satisfied with their choice of this type of therapy, even when they initially hesitated about its effectiveness: “I had doubts about whether it was enough. I saw it worked”. A few other parents criticized the type of therapy their child received: “I think more is possible” and argued that more was needed beyond the child’s enjoyment of the sessions and that no in-depth work had been done on content and issues significant to the child: “I was frustrated when I saw that things weren’t moving, it made me wonder whether art therapy really is something that works”.

### 3.2. Parents’ Perceptions of the Child’s Experiences in Therapy

#### 3.2.1. The Therapy Was Significant for the Child and Provided a Sense of Success and Enjoyment

Some parents considered that their child had a very positive experience in therapy. These parents described the bond between the child and the therapist as characterized by trust. They also highlighted the importance their child attached to therapy, which was reflected in the children’s high attendance rate, taking public transportation on their own to go to therapy, sadness if a session was cancelled, and the children’s attempts to contact the therapist even after the end of therapy. These parents also mentioned the satisfaction, the feeling of success and the enjoyment that the children derived from art making: “She felt comfortable there so that’s what made her continue. If she wasn’t enjoying herself, she wouldn’t want to continue”.

#### 3.2.2. At Times Therapy Elicited Conflict and Resistance in the Child

Some parents stated that their child initially did not want to engage in therapy, which they ascribed to suspicion of the therapist as a result of a history saturated with diagnoses and treatments. This resistance was expressed in refusals to go to therapy: “She just voted with her feet. She did not cooperate. She did not want any treatment”. Some parents noted that even during therapy their child had to deal with difficulties, including the stigma considering that therapy is intended for crazy and problematic children. This caused the children to hide the therapy from their friends, try not to be seen going to therapy, and to resent the mutual transfer of information between the therapist and the educational system: “She really hid it, she didn’t want anyone to know. She took it as something that is only for people who have problems, not for ordinary people”. Some children had trouble sharing content from their world or feelings during therapy: “He was not ready to talk about what he was going through”.

#### 3.2.3. Establishing the Therapeutic Relationship Emerged as a Complex Process, Whose Success Depended on the Therapist’s Professionalism and Positive Character Traits

Some parents described the building of the relationship between the therapist and the child as a long, gradual, complex process: “There were many ups and downs, there were crises”. Some parents explained that to build the relationship, the therapist gave the client control and the right to choose: “She didn’t decide for her. She didn’t tell her come on this day at this time [but] ‘I’m waiting for you and if you choose I’m here for you”. These parents also emphasized the therapist’s strictness about the setting and the rules of therapy: “Consistency. Stability. She gave him the feeling that the sessions were very important and time is very valuable”. These parents primarily described the therapist’s qualities and characteristics which they believed helped establish the relationship with the child, such as empathy, eliciting enthusiasm from the child and focusing on strengths and positive actions.

### 3.3. Implications of Environmental-Social Factors on the Parents’ Perceptions and Experiences of Therapy

#### 3.3.1. Dealing with Stigma

Some parents stated explicitly that among the ultra-Orthodox, anything that deviates from the norm is stigmatized, including therapy. These parents explained that the fear of stigma leads to concealment: “Trying to sweep under the rug or embellish things”, so that severe and complex issues, such as sexual abuse, are not disclosed outside the community or even outside the family. These parents noted that among the ultra-Orthodox there is a widespread perception that therapy is for people with problems and for the insane. In their view, the concealment stems from a fear of tarnishing the good name of the child and the family, and damaging matchmaking—which is of crucial importance for the ultra-Orthodox: “People are afraid of matchmaking. Life is driven by matchmaking. What others will say, what they will see”. A few parents insisted however that there has been a change in the ultra-Orthodox perception of therapy and stated that alongside changes in the relationship between parents and children, getting professional therapy for children has also become more acceptable in the ultra-Orthodox community and is often perceived positively: “Most people today prefer to open things up. Our parents were a little distant from us. We are closer. The world is changing”.

Some parents said that stigma prompted them to hide therapy from their community: “It was very important to me that no one would know”. A few parents even sided with the view that therapy indicates problematic behavior and oddness. By contrast, some other parents emphasized that they strongly opposed social stigma: “When I can, I refute this injustice that is done to children for the sake of phony respect and because of the stigma”. These parents supported therapy and explained that their position stemmed from making the child’s well-being a priority: “First of all, I want the best for my children, I don’t look at what others think of me”. Nevertheless, even parents who opposed the stigma believed that they do not represent the majority in the ultra-Orthodox community, since most of them described what set them apart from mainstream ultra-Orthodoxy, for example, working in the fields of aid and welfare, their Anglo-Saxon backgrounds, or being Baalei Teshuva, which helped explain “maybe that’s why it seems different to me”.

Parental attitudes towards social stigma affected their conduct with their children toward therapy and the explanation they gave to their children for going to therapy. For instance, some parents whose attitude was characterized by criticism of stigma and concealment clearly explained the therapeutic process to their child (even without explicit use of the word “therapy”), by describing the therapy as a place where the child would work on feelings and difficulties, for a better future: “We gave her the feeling that it was the right thing for her”. The few parents whose attitude was characterized by concealment and fear of stigma were reluctant to be transparent and did not tell their child about the role of therapy, fearing that this would lead to a negative self-concept: “We never talked about it with him, I’m afraid to say something that would sound bad to him. You cannot give the child the feeling that something is damaged”. Instead, these parents chose to emphasize the enjoyment of therapy, calling the sessions “classes”: “He goes to class, it’s not therapy—which is problematic”. These parents noted the importance of getting therapy at an early age, before the child understands and absorbs the stigma: “We took him at an early age because later it is more difficult, the child understands that he is actually problematic, he is undergoing therapy, which makes him strange”.

#### 3.3.2. The Relationship between the Parents and the Therapist with the Ultra-Orthodox Education System Emerged as Complex but Very Meaningful for the Parents

Some parents claimed that the ultra-Orthodox education system, which in their opinion has a significant impact on therapy, is not adapted to contain emotional difficulties. They explained that most teachers concentrate on learning, and if a student has problems the teachers usually expect medical treatment or parental guidance in an external setting: “‘I don’t know what to do, he jitters all the time, give him some Ritalin’. That’s how rabbis talk”. These teachers tend to take a behavioral approach—“They use practical language. They want results”, and cooperation between teachers and parents or therapists is rare. According to these parents, the help that the school offers in the case of emotional difficulties usually includes referral to an educational counselor, the school psychologist or remedial teaching. Parents tend to request arts therapies: “This was not the school’s suggestion. I came up with the idea of art therapy and they were a little shocked”.

A few parents emphasized that there is a connection between academic success and the child’s emotional state. These parents explained that educational gaps lead to frustration and damage to self-concept, and therefore affect the child’s behavior, as well as conduct in interpersonal relationships and can account for somatic reactions. In their opinion, therapy can promote academic success by strengthening the child’s self-confidence. One parent noted that academic proficiency is crucially important among the ultra-Orthodox, since it shapes and influences the child’s future in terms of the family (a “good” marriage) and socially: “In good a yeshiva [i.e., school for higher religious education for boys] there are good guys, in the less good Yeshivas the students are kids who dropped out. It’s terribly dangerous”.

The tension between the importance of academic success and the system’s attitude towards emotional difficulties and therapy places parents in a complex situation. A few parents described feeling disappointed, hurt and exhausted, and a few other parents stated that they avoided disclosing the therapy to the educational system: “I was mainly worried about the school. You don’t want him to be labeled as problematic now”. While a few parents described explicit communication with the school system, a few other parents described how the therapist helped them be in contact with the school, including a briefing from the therapist to lay the groundwork for dialogue with the educational staff or a direct, defensive stance on the part of the therapist: “She stood by us throughout this process and talked to the principal and the educational counselor”. However, it is important to note that these parents were referring to an ultra-Orthodox therapist, since in their view they are the only ones who can understand the conflict and the steps required to deal with the education system: “When you know the material, you know how to give good advice, who to talk to”.

Nonetheless, some parents said that even in cases where the therapist was not ultra-Orthodox, she contacted the educational staff, sometimes in a routine and regular manner and sometimes when a problem came to the surface in therapy or in the classroom. A few parents stated that this was the only way the therapist can see the child as a whole and understand social functioning, and explained that ongoing contact between the therapist and the educational system optimizes the therapeutic process: “I think the secret of success is real cooperation between the parties who take care of the child, the school and the parents”. According to a few parents, in their cases the therapist’s attempts to contact the system failed. In a few rare cases the teacher initiated and maintained the relationship with the therapist.

### 3.4. Effects of Intercultural Aspects on Therapy

#### 3.4.1. Cultural/Religious Differences between the Therapist and the Client Elicited Reactions Related to Dealing with the Difference, but Was Accepted for the Benefit of the Client

Some parents described their children’s reactions in cases where the therapist was not ultra-Orthodox. These included the children asking the therapist or the parents about the therapist’s dress code or the child explaining to the therapist about key cultural and religious elements. At the same time, most parents stated that the cultural difference did not bother them, and emphasized the importance of the therapist’s professionalism. These parents explained that the child’s welfare was prioritized over cultural differences: “For me, it was a price I was willing to pay, so that my son would have a little easier time”.

Some parents stated that the best way to deal with the differences in religious observance between them and the therapist was to explain the difficulties that derive from the disparity. Most of these parents stated that they went about explaining politely, but a few took a more interventionist approach which was manifested by their presence during the sessions to monitor and warn the therapist when what was happening did not conform to the family’s norms. A few parents mediated the issue of religious difference with their children, for example by explaining that the main thing is that the therapist was good, while clarifying the difference: “It’s them and it’s not us. We are different and that’s okay”.

#### 3.4.2. Most Parents Preferred an Ultra-Orthodox or Religious Therapist, but Some Compromised on Therapy from a Secular Therapist in an Ultra-Orthodox Setting

Most parents stated that if they had a choice, they would prefer a therapist from an ultra-Orthodox/religious background: “If not ultra-Orthodox, at least religious”. They explained that they compromised when agreeing to therapy from a secular therapist since the sessions took place in an ultra-Orthodox institution: “It was important to me that the environment be ultra-Orthodox”.

Some parents explained that they needed to be sure that the therapist would respect the cultural norms and the content that may come up in the therapy, and would not express overt or covert criticism of the parents: “I feel safer in terms of the attitude, the content, taking a more guarded approach”. A few parents explained that an ultra-Orthodox/religious therapist is able to understand life in the ultra-Orthodox world and the meanings, consequences and nuances which require appropriate consideration in therapy: “There is a difference between a secular child who is in therapy and an ultra-Orthodox child, in terms of needs, difficulties or certain things at home that a therapist with a religious lifestyle can understand better”. A few parents mentioned that children bond better with a therapist with a similar religious/cultural background, since the similarity of speech, dress code and set of values increases their sense of security and closeness: “It is easier for our daughter to connect with people like us”. A few parents stated that an ultra-Orthodox/religious therapist can be a significant figure in their child’s life, since the therapist corresponds to the values they want to impart to their child. Nevertheless, one mother said that a secular therapist was preferable to a non-Orthodox but religious therapist, because sometimes the similarity in religious beliefs (without similarities in cultural affiliation) may lead to complacence, arrogance or wrangling on the part of the therapist.

Some parents explained that if there is no ultra-Orthodox/religious therapist available, therapy should still take place under an ultra-Orthodox setting (such as an institution managed by ultra-Orthodox staff), since the ultra-Orthodox staff can provide guidance to secular therapists on the nuances and terminology, explain what is permitted and prohibited among the ultra-Orthodox, and provide guidance in terms of conduct and content. A few parents mentioned that an ultra-Orthodox setting is advantageous because it can supervise the conduct of secular therapists (including requirements for appropriate clothing and language) and the content that arises in therapy, so that there will be no exposure to content that does not fit with the spirit and values of ultra-Orthodox society: “There is some kind of filter and explanatory system so that there will be no offensive content”. A few parents stated that therapy in an ultra-Orthodox setting increases their sense of belonging and reduces their feeling of being criticized: “It feels like a safer place to put the child, a place that accepts us”.

#### 3.4.3. The Parents Feared a Lack of Understanding by Secular Therapists That Could Undermine Their Children’s Religious Education, and Therefore Requested Restrictions Adapted to the Cultural Norms

Some parents expressed concerns that a secular therapist would show a lack of understanding of their world, including customs, concepts and nuances, the educational system, the lifestyle of ultra-Orthodox society, and the set of values and cultural norms that underlie them. They felt that a lack of understanding on the part of the therapist could make therapy less effective: “If she doesn’t understand all the nuances, it’s less effective”. These parents added that the therapist’s lack of understanding could impede the child’s ability to bond and benefit from the therapist: “This raises many questions for the child. It can undermine trust”. Some parents feared that a secular therapist would have negative attitudes towards the ultra-Orthodox, such as disrespect, arrogance and criticism towards their behavior as parents, their way of life and their way of thinking: “I don’t want anything unwelcoming to infiltrate therapy. Criticism can be suppressed”. Some parents also expressed the fear of exposing their children to prohibited or unacceptable content, which would endanger their children’s Torah and moral education after therapy: “I don’t want the children to hear things that we would not want them to hear, that there will be no harm in the therapy”. These parents emphasized that ultra-Orthodox children are under guidance and supervision: “The ultra-Orthodox child has explicit rules about what is permitted and what is not. The therapists need to be careful not to touch on these topics, not to discuss topics s/he is not familiar with”. These parents explained that their child’s education is at greater risk in therapy, since a close relationship develops with a significant figure: “You develop trust in the therapist. You open your heart. You basically become exposed, so everything the therapist tells you, you accept. This is a very big risk for me”. A few parents did not want their children or themselves to have to interact with a therapist wearing immodest clothing.

Some parents stated that they expected the therapist to stick to “ultra-Orthodox language”: “A more refined, cleaner language. Without slang, without cheap phrases”. In particular, these parents made it clear that the therapist must avoid using explicit words to describe body parts and body secretions. These parents added that the therapist should also adopt appropriate and restrained body language as much as possible: “Calm body language, restrained, not overly physical “. A few parents talked about the importance of adhering to a modest dress code as regards the therapist and the characters used in the therapeutic material. A few parents also referred to the importance of getting guidance from people who belong to the ultra-Orthodox community in general, and from the parents themselves in particular. In their view, the therapist should check whether the techniques used in the therapy are appropriate, and learn from the parents about the unique world of the ultra-Orthodox child and important aspects of life. A few parents stated that boys over the age of 11 should not be treated by a female therapist, or at the very least a rabbi should be consulted: “A teenager and a female therapist is a problem. I wouldn’t do anything without asking a rabbi about this”. A few parents said that computers or cell phone should not be used during therapy.

#### 3.4.4. A Non-Orthodox Therapist Was Seen by Some Parents in a Positive Light: As a Professional Who Enabled Openness and Sharing of Sensitive Content

Some parents stated that despite their concerns, their experience with a non-Orthodox therapist was actually positive. These parents said that they experienced the therapist as professional, culturally sensitive and willing to understand where the family comes from: “She really accepted us, with our opinions, religion, style and conduct”. These parents explained that the therapist respected their lifestyle out of her understanding that it was in the best interest of the client: “She realized that she had to be sensitive to our daughter’s lifestyle, to where she grew up and to the way she was brought up. Not to expose her to things that would confuse her too much”.

Some parents stated that they felt free and open in intercultural therapy precisely because the therapist did not belong to the ultra-Orthodox community: “I’m much more comfortable sharing with someone I don’t know and shouldn’t know”. Some parents suggested that a non-Orthodox therapist has an advantage in handling sensitive and complex cases (such as trauma), since they feel that only a non-Orthodox therapist can understand and contain the complexity, without prejudice or criticism: “I’m not sure that I would feel free enough to go to an ultra-Orthodox therapist and share this with her, because I’m not sure she would understand these things”. A few parents added that the client can also feel more liberated with a non-Orthodox therapist: “He felt more liberated. I did not want my son to feel like he had to hide something”. A few parents felt that in many cases a secular therapist was more professional than an ultra-Orthodox therapist: “It is difficult to find someone in our sector who is a really good, responsible and professional therapist”. These parents added that a secular therapist may have greater openness and flexibility of thought than an ultra-Orthodox therapist: “She doesn’t see things as rigidly as in the ultra-Orthodox sector”.

### 3.5. Perceptions of the Use of the Arts in Therapy

#### 3.5.1. The Parents Recognized the Benefits of Integrating the Arts in Therapy

Some parents explained that the arts are particularly suitable for therapeutic work with children, because psychotherapy that is only verbal is tedious and too abstract for children: “When you sit down to talk with a child, it is very boring. It’s something that should be more experiential”. These parents added that they chose arts therapies based on their child’s interests: “My child really likes creating, really likes to mess with materials, so we came here”. Some parents explained that the arts provided a significant tool for self-expression and noted that their child used the arts to express his loves and desires, as well as to release negative emotions in times of difficulty, in a free and authentic way: “It is a way to express herself without judgment or rules”. They noted that the arts allowed for the expression of content that could not be expressed verbally: “It allowed the expression of places in the soul where there was no speech, there was perhaps not even an awareness or there was no emotional, verbal ability to say them”. Some parents felt that art-making made their child calmer and more open, and as a result, the therapist and the parents were able to establish a relationship with the child and have a meaningful and in-depth conversation: “When engaged in artwork, the child is more liberated, more open, more sharing”. Some parents emphasized that the artwork itself allows others to see what preoccupies the child and reflects the inner world, including processes the child goes through and unconscious content: “It’s an amazing thing, this power of really reading a child’s mind. You can go down very deep with art therapies. It’s like you came to the soul through the back door”. Another advantage was the strengthening of self-confidence through experiences of success: “It gave him a lot of confidence, because he experienced success. He really made very beautiful things”. These parents noted the possibility of empowering the child by presenting his/her works to others. A few parents stated that through engaging in the arts, practical therapeutic goals can be achieved, such as working on playing skills, understanding social situations and problem solving, anger management, flexibility and boundaries.

#### 3.5.2. The Integration of the Arts in Therapy Was Seen as Legitimate and Did Not Conflict with the Religious Views and Cultural Affiliation of the Parents

**Figure 1:**
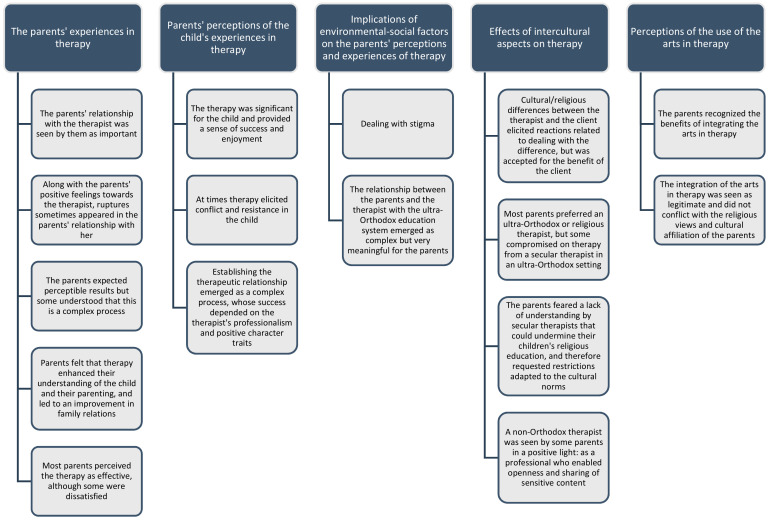
Central domains and core ideas.

Some parents considered that the connection between the arts and therapy is natural and desirable, and that engaging in the arts has become very acceptable in their community. Some emphasized that engaging in the arts is an integral and inherent part of their spiritual views, since the arts connect and bring the individual closer to God: “I think this is a way to worship God. It doesn’t conflict. This is the thing that most causes closeness. The most connecting”. A few parents stated that there was no problem with the use of arts in therapy since it is not real art. These parents explained that in arts therapies the child is involved in simple arts, at their level. However, if they were being instructed in classical and professional artistic work, they would see it as a problem: “It’s not masterpieces, it’s simple things. If, for example, it reached the level of sculpting people or talking to him about all kinds of artists, that would be something else”. Another parent explained that there is no problem with the integration of the arts in the therapy simply because it is child therapy, but if it was art therapy with an adult there would be a problem, because it might lead to expressing forbidden drives: “In child therapy there shouldn’t be a problem. In the treatment of an adult, this can get to inner cravings that are expressed in the artwork and this is a problem”. A few other parents did not understand why there would be a problem in integrating arts in therapy, since there is no connection between the arts and religion: “It’s two different levels”. A summary of the central domains and core ideas appears in Figure 1.

## 4. Discussion

The current study examined the perceptions of ultra-Orthodox parents towards their children’s ongoing arts therapies. This study complements a previous work that examined art therapy for ultra-Orthodox adults [34] and publications that have examined arts therapies for ultra-Orthodox children from the therapists’ points of view [7,8,9].

### 4.1. Understanding the Parents’ Experiences and Perceptions of Therapy from a Socio-Cultural Context

The findings suggest that the participating parents’ experiences were influenced by the ultra-Orthodox view of therapy. The participating parents did differ in their opinions as to the stigma associated with therapy in the ultra-Orthodox, but they were all apparently activated by it and had different ways of dealing with it. For example, some of the participating parents tended to hide the fact that their children were in therapy out of fear of negative repercussions. This finding is consistent with the literature that describes the fear in ultra-Orthodox individuals of having to admit a disability or difficulty, which may create a negative stigma and negatively affect areas of the individual’s life and that of the family [31,32]. It can be assumed that the complexity emerging from the findings as to the experience of ultra-orthodox parents who send their child for arts therapies is related to the characteristics of the ultra-Orthodox community, which is described in the literature as a collective culture in which the community plays a central role in the individual’s life [12]. The social context apparently added further complexity to the parents’ already complex experience, since significant difficulties in a child may overwhelm parents with difficult emotions such as anger, frustration, shame and guilt, such that parents may perceive seeking out therapy as an indication that they are ‘bad parents’ or unable to cope [39]. Nonetheless, some other parents who participated in the current study resisted this stigma and expressed acceptance of therapy. These attitudes may reflect the changes taking place in ultra-Orthodox society, that are multifaceted and characterized by shifts alongside the maintenance of past perceptions of therapy [7,27].

The ultra-Orthodox education system also emerged as another social factor that contributes to the complexity of the experience. The parents who were interviewed in the current study often stated that they were torn between the importance they ascribed to academic success, which in their view plays a decisive role in shaping their child’s family and social future, and the attitude of the ultra-Orthodox education system towards emotional difficulties and therapy, which the participating parents described as non-containing and stigmatizing. Emphasizing the importance of the educational factors in the process from the perspective of the parents who participated in this study, and the impact of the educational system’s perception of the therapy on the parents, contributes to findings from a previous study that described the helplessness of ultra-Orthodox parents in terms of the therapy options offered through the educational system, as well as their preference to send their children for therapy within the school or to outside non-conventional services [33]. Examining the perspective of the educational staff with respect to the therapy that children receive is essential, given their influence on children and the need for cooperation with them to optimize therapy [56]. In light of the centrality of the educational factors in the process according to the parents, and given the lack of research examining the perception of these factors on the arts therapies of ultra-Orthodox children, their point of view should be examined in a follow-up study.

The participating parents also described a technical complexity which is cultural in origin, in the form of barriers that made it difficult for the parents to be present during therapy related to child raising and job obligations, and the financial cost of the sessions. This is probably related to the large numbers of children in the family, high poverty rates and low income characterizing the ultra-Orthodox despite the growing number of men and women in the labor market [13]. This helps shed light on the impact of culture on the ability to engage in therapy, and suggests that intercultural therapy not only involves differences in values and perceptions, but also concrete cultural obstacles that require attention.

Many of the participating parents took a purpose-oriented attitude towards therapy. This attitude is also found in the general population, and it is expressed, for example, in wanting therapy to provide a quick-fix solution to their children’s problem once and for all [39]. However, in the present case this purpose-oriented position tended to dominate. The participating parents focused on the success of the therapy and expected that it would lead to targeted and practical immediate results. Some parents emphasized that the progress should be visible and wondered whether the therapy worked when the change was not noticeable. Most of the participating parents described therapy as effective, but stated that the “problem” that initiated the referral was not completely resolved. Similar to previous descriptions by arts therapists who treat ultra-Orthodox children of many decisions to abridge therapy [7], the participating parents indicated that they did not consider halting treatment to be problematic. A position similar to the participating parents’ perception was also reported in a study on ultra-Orthodox therapists and clients, who described the purpose of art therapy as aimed at correcting failures and improving functioning through the acquisition of tools [34]. It is possible that the purpose-oriented attitude, which also exists among parents in general and is apparently widespread among the ultra-Orthodox, implies a lack of awareness of the meaning of psychotherapy, which may be due to the fact that openness and involvement in psychotherapy in ultra-Orthodox society is a relatively new phenomenon [27], intertwined with other processes of change occurring in this society in the last two decades [13]. This attitude may be related to the characteristics of ultra-Orthodox culture, since according to a widespread claim in the literature, culture also affects self-perception and the individual’s perception of what therapy is [57,58]. These findings may emphasize the importance of the collective in the individual’s experience in ultra-Orthodox society, in terms of the parents’ experience and perception of therapy. As shown in the literature, in ultra-Orthodox society, the self derives its meaning from its belonging to the community and its identification with its goals [59]. Self-fulfillment is expressed, to a large extent, in the realization of the individual’s social vocation [20], and mental well-being. Optimal functioning is linked to a strong sense of relatedness of the individual to the community [60]. As a result, individuals in collective societies may perceive the goals of therapy in a completely different way than therapists endorsing Western concepts, as focusing on solving practical problems without reference to internal change [61]. However, while some participating parents stressed quick results, other parents came to appreciate the processual nature of the therapy and the therapeutic relationship. This may be further evidence of the process of change taking place in the ultra-Orthodox society’s perception of therapy.

The current study also points to the importance of the therapist’s relationship with the parents as a means of developing and strengthening the parents’ understanding of what therapy is and what can be expected from it. Most participating parents said that they understood the importance of their involvement in therapy and their relationship with the therapist, and that they would not interfere or limit the process even if it was not to their liking. A few parents who were interviewed even expressed a willingness to go through a process of change themselves. Furthermore, the participating parents emphasized the progress the therapy brought about in their understanding of their child. The feeling of a greater ability to understand their children and the impact of the parents’ conduct on them after art therapy also emerged from another study in which the perceptions of a non-Orthodox mother were presented [62]. It can be assumed that this is the result of the therapists’ work with the parents. For example, a study on arts therapists who treat ultra-Orthodox children reported that they explain what therapy is, highlight the gradual process, and work to strengthen the parents’ ability to observe and understand their child [7].

### 4.2. Intercultural Aspects in the Relationship between the Parents and the Therapist and in the Therapeutic Relationship

The interviewed parents noted the challenge of dealing with differences in religious affiliation between the therapists, the children and their families, since most arts therapists are not ultra-Orthodox. The parents in the current study talked about their concerns about engaging their children in therapy with a secular therapist, which involves a close relationship with a significant figure who does not share their worldview. The participating parents also emphasized the fear of exposing their children to forbidden or unacceptable content, which they felt could undermine their children’s religious and moral education. A previous study also noted the fear of ultra-Orthodox parents that a non-religious therapist would challenge their spiritual and cultural outlook [33] and lead to ‘spiritual harm’, consisting of a decrease in adherence to the commandments, a violation of social/cultural rules and norms, and deterioration in spiritual faith and the sense of connection with God [63]. These findings are consistent with the literature on the complexity that characterizes intercultural therapies [24] and shows the complexity of the intercultural and value encounter that also exists at the social level.

Even though most of the participating parents said that they accepted the cultural differences between themselves and the therapist because they focused on professionalism and the well-being of their children, they emphasized their preference for an ultra-Orthodox/religious therapist by explaining that their acceptance of a secular therapist was predicated on the fact that the therapy would take place in an ultra-Orthodox setting. Studies have described the tendency of ultra-Orthodox society to rely on internal systems as a way to deal with distress, as well as the preference of certain rabbinic leaders for therapy provided by ultra-Orthodox professionals who acquired their training in secular academic systems [59] and of ultra-Orthodox parents to get assistance from within the community [43]. The participating parents indicated their fear of criticism and negative perceptions from a secular therapist, the fear that a secular therapist would not understand the repercussions and that it would be easier for their child to bond with a therapist from a similar religious/cultural background. It is interesting to note that a previous study that examined the perspective of non-Orthodox therapists on arts therapies of ultra-Orthodox children presented a similar experience to that of the parents who participated in the current study. For instance, that study found that the non-Orthodox therapists who were interviewed indeed acknowledged their criticism of certain aspects and characteristics of ultra-Orthodox society, and stated that their lack of familiarity with concepts and shades of meaning created gaps in the therapeutic relationship, which sometimes made it difficult for the child clients to develop trust [7]. The parents here mentioned the need for an ultra-Orthodox setting, under the guidance of ultra-Orthodox staff and supervision of the therapist and the content in the therapy. These feelings about the ultra-Orthodox setting correspond with the experiences of non-Orthodox therapists, who stated that they benefitted from guidance from the parents and the ultra-Orthodox staff, and were supervised by the institute’s management concerning their dress, the content, and the tools used in therapy [7]. However, in another study, non-Orthodox therapists noted that the guidance they received mainly concerned their outward appearance and not how best to deal with the characteristics of the ultra-Orthodox culture, which led to misjudgments on their part in therapy [9].

The participating parents noted the adjustments that should be made in intercultural therapies. These include adapting the therapist’s language to the ultra-Orthodox code (clean and refined language, without the use of slang or explicit words to describe body parts and bodily secretions), adopting a restrained body language, adhering to a modest dress code, receiving guidance from members of the ultra-Orthodox community, avoiding the use of modern technology during therapy, and avoiding assigning adolescent boys to a female therapist. These concrete guidelines may be meaningful and valuable, since clear strategies for overcoming the difficulties that arise in intercultural therapy are rarely presented in the literature, or are vague and difficult to implement [64].

Nevertheless, the participating parents also referred to the benefits inherent to intercultural therapy. Some parents stated that they and their children felt more open and freer to share, precisely because the therapist did not belong to the ultra-Orthodox community. Some parents perceived the non-Orthodox therapist as more able to understand and contain particularly sensitive and complex cases because there was no judgmentalism towards the content being revealed. Previous studies have reported the positive effects of receiving therapy in a neutral and external environment on the increased sense of security and willingness to disclose on the part of ultra-Orthodox parents and clients [7,8,27]. The literature dealing with intercultural therapies also shows that among clients from other traditional and closed societies, such as Arab-Muslims, there may be a preference for receiving therapy from a therapist who comes from a different cultural or religious background, out of a desire for secrecy and in order to avoid revealing personal secrets in the community to which the clients belong, as well as fear of being judged [65]. In addition, a few parents who were interviewed also stated that a secular therapist would be more professional and have greater openness and flexibility of thought than an ultra-Orthodox therapist.

### 4.3. The Perception of the Meanings of the Use of the Arts in Therapy

The participating parents did not experience conflicts with the use of the arts in therapy. These parents listed various benefits, including adaptation to child therapy, the possibility of authentic non-verbal self-expression, the possibility of creating a relationship and conversation through artistic work, observing the child’s inner world through creation, and strengthening self-confidence through experiences of success. The parents made it clear that the nature of the arts integrated into therapy did not pose a threat to them, and some emphasized that the connection between the arts and the therapy is natural and desirable. These findings run counter other studies that have examined arts therapies in ultra-Orthodox society, in which it was suggested that the encounter between the arts, religion and culture may involve the fear of violating the religious prohibitions on idol making, a fear of creative expression that is not modest, as well as difficulty in playfulness that evokes feelings of anxiety and guilt [7,34]. This may be related to the difference between therapy for children and therapy for adults. As explained by the participating parents, the arts in which the children engage in therapy are simple arts appropriate to their level and therefore do not pose a risk of forbidden inclinational expression. Alternatively, the differences may stem from observing a cultural phenomenon from the point of view of individuals who do not belong to the culture and observing the phenomenon from the point of view of the members of the culture themselves, in this case the ultra-Orthodox parents. Perceptions on the part of outsiders may be influenced by their views of society and a set of values they do not share. On the other hand, social desirability biases may lead members of the culture to present themselves in a positive light, which does not necessarily correspond to their true feelings [66].

However, the participating parents’ comments suggested that some of them did not understand the connection between artistic activity and an improvement in their children’s behavior. For example, some parents felt that the therapy focused on enjoyment, but that significant and in-depth therapeutic work did not take place, since they felt that this must also include a verbal reflection of the unspoken content that arises in the artistic work. This showed a lack of familiarity or understanding of the meaning and power of artistic work in therapy. As described in the literature, the arts can be a language for complex content which is forbidden or prevented from reaching verbal consciousness, or content that cannot be communicated through words [67]. Furthermore, symbolic artistic work, which is not necessarily verbal, is essential to the healing process. It provides a safe, distant and non-verbal medium for expressing a difficulty, observing it and discovering alternatives for a solution, thus enabling the clients to carry out internal integration and make changes in their own lives [68]. It is worth noting that criticism of arts therapies and the parents’ lack of understanding of the basis and essence of arts therapies is also true for the general population, such as parents of children with autism [69].

### 4.4. Implications for Clinical Practice

The findings suggest that in intercultural therapy in general, and in therapy for ultra-Orthodox children in particular, the therapist’s relationship with the parents is of utmost importance, because this relationship is essential to the parents’ understanding of therapy and their child. The therapist must be aware of the social pressure that some parents and children face as a result of being in therapy, the gaps in understanding of therapy and its goals among ultra-Orthodox individuals, and take steps to make the therapy more accessible. The ultra-Orthodox parents who participated in this study tended to perceive therapy in a purposeful way. This suggests that therapists’ work with parents should also deal with practical and focused goals and expectations, and provide a psycho-educational explanation of the ways in which arts therapies can affect the achievement of these goals. Special attention should be paid to the therapist-teacher relationship, with an emphasis on careful communication that promotes understanding of therapy and empowering the parent in this relationship. The findings show that the therapist in intercultural therapy, especially for ultra-Orthodox children, must receive guidance from members of the culture and adapt to the culture’s characteristics and priorities. Consideration must be given to intersectionality and transferences originating from intercultural differences. The findings also show the importance of training ultra-Orthodox therapists, and establishing dedicated professional therapy centers for the community through cooperation with members of the culture.

### 4.5. Limitations of the Study

The current study sought to examine the arts therapies of ultra-Orthodox children from the perspective of the parents of the children in treatment. Since the few studies that have examined arts therapies in ultra-Orthodox children have focused solely on the perspective of the therapists, the current study enables an initial observation of the experience of fathers and mothers who belong to all factions of ultra-Orthodox society with respect to arts therapies for their children. However, there are several limitations to this study. All the interviews were conducted over the phone. Telephone interviews had substantial advantages for the participants including the convenience of providing sensitive information and extended access to participants, especially individuals who are difficult to contact such as mothers of small children or members of closed religious communities. However, one disadvantage of this technique is the reduction of social cues, and in particular the inability to use information from the interviewee’s body language. Another disadvantage is that the interviewers had fewer opportunities to create a good interview climate because they could not see the interviewee’s context. Unlike face-to-face interviews, the interviewee may be in the presence of others during the interview and be interrupted [54]. In addition, the children of the parents interviewed in this study received various types of arts therapies (including visual art therapy, dance/movement therapy, bibliotherapy, music therapy and psychodrama). It is possible that focusing on examining one type of therapy would have enabled a deeper understanding of the specificity of different modalities of arts therapies and the differences in their possible impact on the therapy of ultra-Orthodox children. Another limitation is related to the fact that the interviewed parents referred to therapies provided in different settings (including an ultra-Orthodox institution, a school, a public clinic and private clinic). This diversity may have influenced and shaped the experience, and raises questions about the influence of other untested variables on therapy.

### 4.6. Suggestions for Future Research

This study complements a previous study which examined therapy from the perspective of non-Orthodox arts therapists. However, to achieve a broader and more comprehensive understanding, further research is needed which should examine the perspective of the educational staff and the perception of the children themselves. In order to understand specificity and possible variations in the influence of the different types of arts on the therapy of ultra-Orthodox children, further research should focus on modality. Similarly, future research should examine the therapy delivered to ultra-Orthodox children in one single setting (for example in one ultra-Orthodox institution or in the educational system). Further research could also delve into possible differences between the various factions in ultra-Orthodox society in terms child arts therapies. In addition, a follow-up study involving the collection of quantitative data through an attitude survey tapping the perceptions and experiences of the parents could provide a clearer picture of the prevalence of the experiences described in this study and help evaluate development and changes over time.

## Figures and Tables

**Table 1:** Demographics.

Parent Gender	Parent Age Range	Number of Children in the Family	Faction	Client Gender	Age of the Client When Receiving Therapy (in 5-Year Age Brackets)	Months of Therapy	Type of Therapy	Therapeutic Setting	Religious Affiliation of the Therapist
Male	31–40	8	Hasidim	Girl	6–10	13–24	Bibliotherapy	Public clinic	Religious Zionist
Male	51–60	5	Hasidim	Boy	–	37–48	Visual art	Haredi institution	Secular
Male	31–40	3	Lita’im	Boy	1–5	1–12	Visual art	Haredi institution	Secular
Male	31–40	4	Lita’im	Girl	1–5	1–12	Music	Haredi institution	Ultra-Orthodox
Male	21–30	4	Sephardic Haredim	Boy	1–5	13–24	Visual art	Haredi institution	Secular
Female	21–30	4	Lita’im	Boy	1–5	25–36	Dance/ movement	Haredi institution	Secular
Female	31–40	7	Hasidim	Girl	11–15	13–24	Psychodrama	Haredi institution	Religious Zionist
Female	41–50	5	Baalei Teshuva	Girl	6–10	37–48	Dance/ movement	Haredi institution	Secular
				Boy	6–10	25–36	Psychodrama		Religious Zionist
Female	31–40	1	Lita’im	Boy	6–10	25–36	Visual art	Haredi institution	Religious Zionist
Female	-	7	Hasidim	Boy	11–15	1–12	Visual art	Haredi institution	Religious Zionist
Female	31–40	8	Lita’im	Boy	6–10	13–24	Visual art	Private clinic	Religious Zionist
Female	21–30	4	Lita’im	Girl	6–10	1–12	Dance/ movement	Haredi institution	Secular
Female	41–50	5	Hasidim	Boy	6–10	25–36	Visual art	Haredi institution	Secular
Female	31–40	5	Lita’im	Boy	6–10	13–24	Music	Haredi institution	Ultra-Orthodox
Female	31–40	4	Hasidim	Girl	6–10	49–60	Visual art	School	Ultra-Orthodox
Female	31–40	6	Hasidim	Girl	11–15	1–12	Visual art	Haredi institution	Secular
				Girl	6–10	1–12	Bibliotherapy		Secular
				Boy	6–10	13–24	Bibliotherapy		Secular
				Boy	1–5	13–24	Visual art		Secular
Female	31–40	6	Baalei Teshuva	Boy	1–5	1–12	Dance/ movement	Haredi institution	Religious Zionist
				Girl	1-5	1-12	Bibliotherapy		Secular

## Data Availability

The datasets for this manuscript are not publicly available to protect participants’ confidentiality. Requests to access the datasets should be directed to L.K., lali.livyatan@gmail.com.

## Appendix A. Interview Guide for Parents of Ultra-Orthodox Clients

1. **The encounter between ultra-Orthodox parents and children and psychotherapy**
  1.1 Tell me what prompted you to seek therapy for your child.
  1.2 What brought you to therapy and what made you stay in therapy?
  1.3 What are the most important things you expect from therapy for your child?
  1.4 What, in your opinion, are the goals of arts therapies?
  1.5 Are there differences between the way you see the goals of therapy and the way the therapist sees them?
  1.6 What are the main dilemmas you face as a parent of a child receiving therapy?
  1.7 What content would you like to be included in the therapy? What content should be avoided in therapy?
  1.8 Are there consequences to your decision to seek therapy for your child? If so, which ones?
  1.9 Does your community know that your child is receiving therapy? How do you feel about that?
2. **Arts therapies in ultra-Orthodox society**
  2.1 What is art (or drama/music/movement) for you?
  2.2 What do you think should be the role of art (or drama/music/movement) in therapy?
  2.3 Do you think art (or drama/music/movement) promotes therapy? How?
  2.4 How do you see the relationship between therapy, art, culture and religion.
  2.5 Arts therapies can stimulate emotional expressions, playfulness and release. What is your attitude towards this?
3. **The intercultural encounter**
  3.1 Is the cultural/religious background of the therapist important to you?
  3.2 If you could choose, would you prefer your child’s therapist to be from a similar or different cultural background to yours? Why?
  3.3 Do you feel that the cultural difference between therapist and client has an effect on therapy?
  3.4 Do you think cultural similarity between therapist and client has an effect on the therapy? In what ways?
  3.5 What do you think could help bridge the cultural gap between a non-Orthodox therapist and an Orthodox client?
  3.6 What, in your opinion, are the advantages and disadvantages of working with a therapist who comes from a cultural background similar to the client?
  3.7 What, in your opinion, are the advantages and disadvantages of having a therapist who comes from a different cultural background than the client?
